# A New Application of the Electromagnet

**Published:** 1902-12-27

**Authors:** 


### A NEW APPLICATION OF THE ELECTRO-MAGNET.

A very useful application of the electro-magnet guided by the X-rays for the extraction of magnetisable foreign bodies from the stomach has been described by Stephen Mayou, F.R.C.S.[1] A boy somewhat over two years old swallowed a hair pin, and examination by the X-rays showed the shadow of the pin just to the right of the middle line, below the liver shadow, in a vertical position, apparently across the pyloric orifice. There were no symptoms. Two days later it was still in the same position, only lying horizontally with the blunt end towards the duodenum, into which the following day it had apparently passed. Seven weeks later it appeared not to have further changed its position, and was still lying to the right about the level of the umbilicus. The case was then handed over to Mr. Mayou, and an attempt was made, fortunately with success, to extract the foreign body by means of an electro-magnet guided by X-rays. The electro-magnet, which was made for the purpose, was about two inches long, and had a lifting power of a quarter of a pound when connected with a four-volt battery. This magnet was inserted into an ordinary stomach tube with the end cut off, or preferably it might be inserted into a tube with a perfectly smooth inner surface so that the magnet could be glided up and down with ease by pulling on the stiff wires by which it is connected with the battery. A narrow silver band was placed on the outer surface of the stomach end of the tube so as to render its shadow visible under the X-rays. The patient was anæsthetised and the tube containing the magnet was passed down the œsophagus into the stomach, this being accomplished with some little difficulty on account of the magnet being too long to take the pharyngeal curve easily. When it arrived in the stomach the magnet was protruded about half an inch from the tube, the room was darkened, and the rays were turned on. Both magnet and hairpin were plainly seen and the current was then allowed to flow round the electro-magnet. This produced adhesion between the magnet and the hairpin, which latter was then drawn into the tube and safely extracted, the tube, magnet and hairpin being withdrawn together.

[1] Lancet, Dec. 6.

